# Genotyping by sequencing for genomic prediction in a soybean breeding population

**DOI:** 10.1186/1471-2164-15-740

**Published:** 2014-08-29

**Authors:** Diego Jarquín, Kyle Kocak, Luis Posadas, Katie Hyma, Joseph Jedlicka, George Graef, Aaron Lorenz

**Affiliations:** Department of Agronomy and Horticulture, University of Nebraska, 363 Keim Hall, Lincoln, NE 68583 USA; Institute of Genomic Diversity, Cornell University, Ithaca, NY USA

## Abstract

**Background:**

Advances in genotyping technology, such as genotyping by sequencing (GBS), are making genomic prediction more attractive to reduce breeding cycle times and costs associated with phenotyping. Genomic prediction and selection has been studied in several crop species, but no reports exist in soybean. The objectives of this study were (i) evaluate prospects for genomic selection using GBS in a typical soybean breeding program and (ii) evaluate the effect of GBS marker selection and imputation on genomic prediction accuracy. To achieve these objectives, a set of soybean lines sampled from the University of Nebraska Soybean Breeding Program were genotyped using GBS and evaluated for yield and other agronomic traits at multiple Nebraska locations.

**Results:**

Genotyping by sequencing scored 16,502 single nucleotide polymorphisms (SNPs) with minor-allele frequency (MAF) > 0.05 and percentage of missing values ≤ 5% on 301 elite soybean breeding lines. When SNPs with up to 80% missing values were included, 52,349 SNPs were scored. Prediction accuracy for grain yield, assessed using cross validation, was estimated to be 0.64, indicating good potential for using genomic selection for grain yield in soybean. Filtering SNPs based on missing data percentage had little to no effect on prediction accuracy, especially when random forest imputation was used to impute missing values. The highest accuracies were observed when random forest imputation was used on all SNPs, but differences were not significant. A standard additive G-BLUP model was robust; modeling additive-by-additive epistasis did not provide any improvement in prediction accuracy. The effect of training population size on accuracy began to plateau around 100, but accuracy steadily climbed until the largest possible size was used in this analysis. Including only SNPs with MAF > 0.30 provided higher accuracies when training populations were smaller.

**Conclusions:**

Using GBS for genomic prediction in soybean holds good potential to expedite genetic gain. Our results suggest that standard additive G-BLUP models can be used on unfiltered, imputed GBS data without loss in accuracy.

## Background

Marker-assisted selection (MAS) has played an important role in soybean breeding, particularly for traits that are difficult to evaluate phenotypically such as soybean cyst nematode (SCN) resistance. An early demonstration of successful MAS for SCN resistance allowed accurate identification of resistant lines using microsatellite markers [1]. Use of MAS for improving grain yield, however, has been met with limited success in soybean. A host of QTL mapping studies have reported QTL for grain yield in exotic soybean populations [2–5], but introgression of yield QTL has not been consistent across different genetic backgrounds [6]. Moreover, the QTL mapping – introgression approach is difficult to justify unless large effect QTL alleles are identified, which is rarely the case for grain yield.

Sebastian et al. [7] reported on a MAS approach that involved the sub-lining of existing soybean elite cultivars derived from single F3 or F4 plants. The authors called this approach context-specific MAS. Essentially, MAS is performed within narrow populations (i.e., elite cultivars with residual heterogeneity) with the goal of obtaining more precise estimates of genetic value in early field trials consisting of only a single replication. Lines were selected and advanced for further testing on the basis of marker scores calculated using significant marker effects estimated within populations. Significant superiority in grain yield for some of the selected sublines, relative to their “mother lines” (i.e., the elite cultivars with residual heterogeneity), was demonstrated using this approach [7].

This common-sense approach is ideal for increasing accuracy of preliminary yield tests: marker effects are estimated more accurately because marker alleles are highly replicated across individuals comprising a large population, whereas phenotypic values are estimated using only a single observation. Marker effects, however, can only be applied for calculating marker scores within a single bi-parental population and therefore, the current generation. It would be desirable to predict breeding values after one or more generations of recombination and selection in order to facilitate rapid cycling of parents. Furthermore, pooling genotypic and phenotypic information across populations could allow for more populations to be evaluated for grain yield within the same field space. Fewer individuals could be phenotypically evaluated in each biparental population and the breeding value of remaining (non-evaluated) individuals could be predicted using markers. Current trends strongly indicate plant breeding programs will be limited by phenotyping capacity, not genotyping capacity, thus increasing the attractiveness of this strategy through time.

Genomic selection (GS) has become the predominant method of applying molecular markers for selection of complex traits in plant breeding programs [8]. Briefly, genomic selection entails building a prediction model through associating marker information with phenotypic information in a “model training” step. Individuals that have been genotyped and phenotyped comprise the “training population” or “calibration set”. The prediction model is applied to a set of selection candidates that have been genotyped but not evaluated phenotypically. The primary difference between GS and traditional forms of MAS is that QTL mapping is not performed and markers are not chosen for inclusion in the model based on a statistical analysis, but rather all marker information is used simultaneously. The types of models used to deal with the “large *p*, small *n*” problem created by the genomic approach to prediction have been reviewed and compared elsewhere [8–10].

Dramatic advances in sequencing technologies are providing highly dimensional molecular marker information at low cost. Genotyping by sequencing [11] is a method well described by its name: polymorphisms are scored using next-generation sequencing technologies followed by a bioinformatics pipeline. The advantage of GBS is that it reduces cost through an enzyme-based genomic complexity reduction step and the use of barcoded adapters for multiplexing [12]. Genotyping by sequencing has been applied to investigations of genetic diversity in maize [13] as well as to studies on GS [14–16]. Working in soybean, Sonah et al. [17] developed a novel GBS protocol and reliably called 10,120 high-quality SNPs among eight diverse lines. These authors called high-quality SNPs displaying only a small percentage of missing data, whereas many applications of GBS [16] have tolerated SNPs with very high frequencies of missing data, sometimes up to 80%.

Given this high rate of missing values in GBS data, imputation of marker scores is typically performed. The best imputation method, and whether imputated GBS data provides better predictions than simply selecting SNPs with low rates of missing data, however, is not known. Rutkoski et al. [18] showed a slight advantage to using imputation when markers were used with high missing data rates. In maize, however, Crossa et al. [16] failed to find any improvement in prediction accuracy using a haplotype-based imputation method on GBS data. Poland et al. [14] showed that a random forest imputation method provided the most accurate imputations, but the effect on genomic prediction accuracy was not significant.

A large number of studies on GS in multiple crops has been reported [2, 19–22]. A study on GS in soybean, however, has not. Moreover, there are only a few reports on the use of GBS for GS [14–16]. In light of the current research on GS and the dearth of reported research on GS and GBS in soybean breeding, the objectives of this study were (i) evaluate prospects for GS using GBS in a typical soybean breeding program and (ii) evaluate the effect of GBS marker selection and imputation on genomic prediction accuracy. To achieve these objectives, a set of soybean experimental lines sampled from the University of Nebraska-Lincoln Soybean Breeding Program were genotyped using GBS and evaluated for grain yield and other agronomic traits at multiple Nebraska locations. Reported findings are important to the application of GBS to selection for grain yield in future soybean breeding efforts.

## Methods

### Germplasm and phenotyping

Three hundred and one soybean experimental lines currently in advanced stages of the University of Nebraska-Lincoln Soybean Breeding Program were sampled. Two hundred and seventy-five lines were in the F_5:8_ generation and twenty-six lines were in the F_4:7_ generation. Soybean lines belonged to maturity groups I (N = 64), II (N = 213), and III (N = 24) (Table 1) and represent 34 biparental families ranging in size from one to 28 lines per family. Median family size was 8.

**Table 1:** **Number of lines belonging to each maturity group (MG) and grown at each Nebraska location**

	Beemer	Phillips	Cotesfield	Mead	Lincoln	Clay center
MG 1	64	64	64	64	0	0
MG 2	213	213	213	213	0	0
MG 3	0	24	0	24	24	24
Total	277	301	277	301	24	24

During the summer of 2011, soybean lines were grown in two-row plots (0.76 m apart, 2.9 m long) seeded to a density of 26 seeds per square meter. Plots were arranged in an augmented incomplete block design with two replications. Blocks consisted of 27 – 39 experimental entries and three check cultivars. Lines belonging to maturity groups I and II were evaluated at the Nebraska locations Beemer, Phillips, Cotesfield, and Mead. Lines belong to maturity group III were evaluated at the Nebraska locations Phillips, Mead, Lincoln, and Clay Center (Table 1). Grain yield (GY; Mg ha^-1^) was measured at all locations, plant height (PH; cm) was measured only at Mead, and days to maturity (MD) was measured at Beemer, Phillips and Mead. Grain yield was recorded as machine harvestable grain yield adjusted to 13% moisture. Plant height was measured as the distance (in centimeters) between the surface of the soil and the main-stem apical meristem. Days to maturity was defined as the number of days from planting until the R8 stage when 95% of the pods were mature and brown in color.

Phenotypes were adjusted to remove location and block effects according to the model:


where *g*_*i*_ represents the effect of the *i*^th^ genotype (i.e., soybean line), *e*_*j*_ represents the effect of the *j*^th^ location, *r*_*k(j)*_ represents the effect of the *k*^th^ replicate nested in location *j*, *b*_*l(k)*_ represents the effect of the *l*^th^ incomplete block nested within replicate *k*, and. Best linear unbiased estimates were calculated for soybean lines and input into the genomic prediction models described below. The genotype effect was also treated as random in order to estimate variance components for the purpose of estimating heritability. Broad-sense heritability (*H*^2^) on an entry-mean basis was calculated as  where  is the variance among soybean lines,  is the genotype-by-environment interaction variance,  is the residual variation, and *e* and *r* in this context are the number of environements and replications within environments, respectively.

### Genotyping

Leaf discs were collected from 12 random plants of each soybean line at approximately the V6 growth stage. DNA isolation was performed using the Qiagen DNeasy Plant 96 kit. Samples were sent to the Institute of Genomic Diversity at Cornell University for genotyping by sequencing as described in [11] and at http://www.maizegenetics.net/gbs-overview. Briefly, DNA samples were digested with the ApeKI restriction enzyme followed by ligation of adapters to fragment ends. Adapters consisted of Illumina sequencing primers and a barcode adapter. After adapter ligation, samples are combined into pools consisting of 96 samples. A PCR amplification is carried out to create the GBS libraries, which are submitted to a single Illumina HiSeq2000 flow cell for sequencing. Four sequenced libraries produced on average 247,255,883 reads, of which on average 219,580,690 were good, barcoded reads.

The GBS analysis pipeline as implemented in Tassel Version 3.0.156 was used to call SNPs. Briefly, 1) tag counts were generated from fastq files with the FastqToTagCountPlugin (options: -s 300000000, -c 1), 2) tag counts were merged with the MergeMultipleTagCountPlugin (options: -c 5), 3) tags were aligned to the reference genome Gmax_109_softmasked.fa.gz, which was downloaded from ftp://ftp.jgi-psf.org/pub/JGI_data/phytozome/v8.0/Gmax/assembly/ on 16 July 2012 and indexed for use with bwa version 0.6.1-r104. BWA version 0.7.3a-r367 was used for alignment (-n 0.04). Chromosomes were renamed for compatibility with the GBS pipeline by removing the leading 'Gm’ and 'scaffold_’, and then converted to the tags-on-physical-map format using the SAMConverterPlugin 4). Counts of tags per individual (taxa) were generated with the FastqToTBTPlugin (options: -c 1 –s 300000000, -y), 5) Counts of tags per individual were merged with the MergeTagsByTaxaFilesPlugin (options: -s 300000000, -x), 6) SNPs were called using the TagsToSNPByAlignmentPlugin (options: -mnMAF 0.01, -mnLCov 0.1, -mnMAC 10, -mxSites 1,000,000). Duplicate sites were merged with the MergeDuplicateSNPsPlugin (options: -callHets, -misMat 0.05), and duplicated taxa were merged with the MergeIdenticalTaxaPlugin (options: -hetFreq 0.8). Scaffolds were ignored for SNP calling.

### Genomic prediction models

Base pair calls contained in the hapmap file obtained from the GBS analysis pipeline were converted to numerical genotype scores, *x* ∈ {0, 1, 2}, where *x* is the number of copies of the major allele.

Two genomic prediction models were studied: a standard G-BLUP model including only additive effects, and an extended version of the G-BLUP model also including additive-by-additive effects. Two different formulations of additive-by-additive effects have been presented in the literature [23, 24] and both of them were considered.

The standard G-BLUP model including additive effects only is
1

where *y*_*i*_ represents the phenotype of the *i*^th^ line, *μ* represents the intercept, *g*_*i*_ represents the additive genetic value, and *e*_*i*_ represents the residual. The additive genetic value can be estimated as , where *x*_*ij*_ is the genotype score at the *j*^th^ (*j =* 1,…,*p*) locus in the *i*^th^ (*i =* 1,…,*n*) line, and *b*_*j*_ is the allelic substitution effect (marker effect) at the *j*^th^ marker locus. Marker effects were considered as *IID* random variables from a normal distributions such that . From properties of the multivariate normal distribution the vector **g** = **Xb**, (**g** = [*g*_1_, …, *g*_*n*_]′), follows a multivariate normal distribution with null mean and covariance matrix  where  and . Hereafter, we centered and standardized genotype scores by dividing by , where *θ*_*j*_ is the estimated allele frequency at the *j*^th^ marker locus. The **G** matrix is a genomic realized relationships matrix whose entries are given by . Summarizing above stated assumptions the model (1) becomes a mixed effects model with  and . Using this model, the lines are related through the off-diagonal values of **G** matrix, allowing the borrowing of information between lines to predict performance of lines not phenotyped.

Additive-by-additive effects were modeled using two different covariance structures among lines. Several authors [25, 26] modeled additive-by-additive epistasis through a **G** ∘ **G** matrix following Cockerham (1954) and Kempthorne (1954), where ∘ represents the Hadamard, or element-wise, multiplication operation. The first model including additive-by-additive epistasis was
2

with  and .

More recently, Xu [24] proposed an alternative way to include these interaction effects using the covariance structure given by , with ,  and *Z*_*k*_ is the *j*^th^ marker locus such that  Using this assumption a different version of the common epistasis model is given by:
3

with  and .

Modeling additive and additive-by-additive components was conducted to assess the proportion of the phenotypic variance accounted for by these effects and improvements in accuracy of genomic prediction. By combining models (1) and (2), a model including additive and epistatic effects was formulated:
4

with ,  and .

The alternative additive and additive-by-additive model following Xu [24] was built combining models (1) and (3):
5

with ,  and .

Models (1)-(5) were fitted to the full data set using computational methods described in [27]. All the statistical analyses were implemented in the R statistical software [28].

### Marker imputation

Genotyping-by-sequencing data sets typically have high rates of missing data [14, 16]. Three imputation methods were considered to impute missing values of the soybean GBS data set. (*i*) Naïve imputation substitutes missing values at each locus with 2*θ*_*j*_, where *θ*_*j*_ is the estimated frequency of the major allele at the *j*^th^ locus. This method is not expected to add information, but rather serves the purpose of ensuring unchanged allele frequencies after imputation and provides a marker matrix containing no missing data so that analytical operations can be performed. (*ii*) Random forest imputation is based on random forest regression introduced by Breiman [29]. Marker imputation for this study was performed using the MissForest R package according to [18]. The algorithm was performed chromosome-wise and for each PMV and MAF combination in parallel. (*iii*) FILLIN (HI) is a novel imputation method based on haplotypes, which is implemented in TASSEL 5.0. Default settings were used with the exception of maximum heterozygosity, which was set to 0.30 using the option -mxHet. Detailed information can be found in the TASSEL 5.0 User Guide at http://www.maizegenetics.net.

### Varying factors affecting genomic prediction accuracy

To evaluate the effects of GBS marker selection and imputation methods on genomic prediction accuracy, two criteria for filtering SNPs were considered. Filtering of GBS SNPs was done sequentially, first filtering based on percent missing values (PMV), and then, for minor-allele frequency (MAF). Levels for PMV (27) and MAF (12) were (*l*) 1*–*20, 25, 30, 40, 50, 60, 70, and 80%, and (*m*) 0.05-0.1, 0.15, 0.20, 0.25, 0.30, 0.35, and 0.40, respectively. Markers were filtered based on all possible combinations of PMV and MAF. After filtering, remaining missing values were imputed using each of the three imputation methods described above. This produced 972 marker datasets (e.g., 27 PMV levels × 12 MAF levels × 3 imputation methods).

All comparisons were made on the basis of the correlation between the observed phenotype and the predicted breeding value, which is referred to as *predictive ability*, following [30]. We reserve the term *prediction accuracy* (*r*_*gp*_) for the correlation between the prediction and the true breeding value. Prediction accuracy can be approximated by dividing predictive ability by  [8, 31]. Predictive ability () of each marker filtering criteria was evaluated using 10-fold cross validation replicated 200 times. The mean predictive ability across the 200 replicates was calculated and bootstrap confidence intervals.

The impact of training population size on prediction accuracy was evaluated using a validation set comprised of 50 randomly selected lines and training sets of variable sizes. The validation set was formed by randomly sampling 50 lines without replacement. From the remaining 251 lines, the training population of size *n* was formed sequentially such that its size ranged between 2 and 251. First, two lines were sampled (i.e., *n* = 2) without replacement, then, from the remaining 251-*n* lines, additional lines were incorporated to the training set, by increments of one. Once a line was sampled, it remained in the training set. The validation set was held constant with the initial 50 lines. Two GBS marker subsets were used to evaluate training population size effect: 1) PMV ≤ 5% and MAF > 0.05; and 2) PMV ≤ 80% and MAF > 0.3. This procedure was repeated 1000 times and accuracies at each training population size were averaged across replicates.

## Results

A total of 5,770,366 unique 64-bp sequence tags were identified across all four soybean libraries, of which 68.75% aligned uniquely to the reference genome, 11.32% aligned to multiple positions and 19.92% could not be aligned. The mean (median) sequencing depth per SNP locus was 11 (6), with mean (median) proportion “missingness” per SNP locus of 0.18 (0.08).

**Figure 1:**
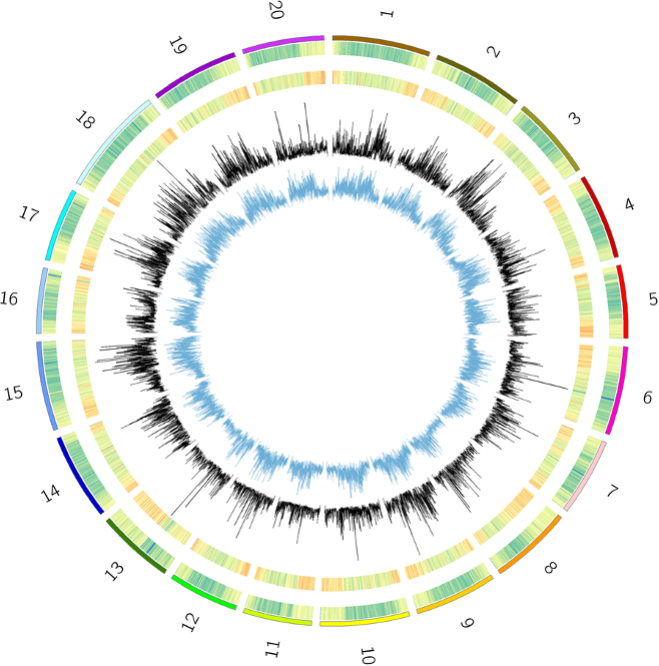
**Genoptyping by sequencing parameters on 301 elite soybean breeding lines.** Parameters were calculated using a 100 kbp window with a 50 kbp slide. From outside to inside: 1) Unique 64-bp sequence tags per window; 2) SNP density; 3) Minor-allele frequency; 4) Percent missing values. For the 64-bp sequence tag and SNP density heatmaps, hot colors represent larger values on a log base three scale.

**Figure 2:**
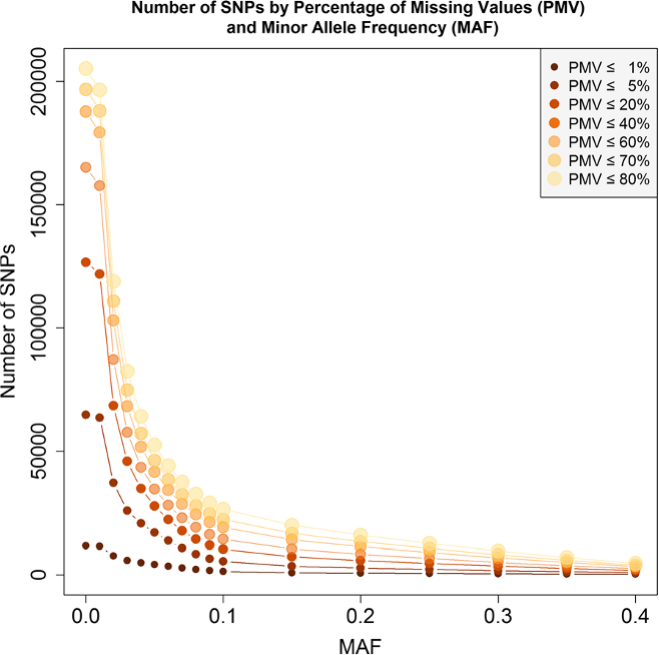
**Number of SNPs remaining after applying filtering by combinations of minor-allele frequency and percent missing values.**

Unique tag counts and SNP density were higher towards the chromosome ends compared to pericentromeric regions (Figure 1). The GBS protocol targets gene-rich regions, such as the chromosome ends, through the use of methylation sensitive restriction enzymes. Related to the distribution of uniqe tag counts, percent missing values were lower towards chromosome ends and higher towards the pericentromeric regions. There was no apparent pattern regarding MAF with the exception of some chromosomes harboring more diversity than others (e.g., chromosomes 11 and 20 versus chromosomes 15 and 18) (Figure 1). The number of SNPs remaining after filtering by MAF and PMV is shown in Figure 2. The number of SNPs available with cuttoff values of PMV ≤ 80% and MAF > 0.05 was 52,349. There were 16,502 SNPs with PMV ≤ 5% and MAF > 0.05.

The high quality of the phenotypic data was reflected with relatively high heritabilities of 0.69 for GY and 0.94 for MD (Table 2). As expected, the genotype-by-environment interaction source of variation was greater for GY compared to MD. The overall  of G-BLUP using a SNP dataset with cuttoff values of PMV ≤ 80% and MAF ≥ 0.05, and the Naïve imputation method, was 0.565 for GY, 0.374 for PH, and 0.644 for MD. Prediction accuracy estimates for GY, PH, and MD were, 0.64, 0.42 and 0.65, respectively.

**Table 2:** **Summary of phenotypic data analysis for grain yield (GY), plant height (PH) and days to maturity (MD)**

Trait	Units	Mean	SD ^†^	Range	Variance component ^‡^			***H*** ^2^
					G	G × E	Residual	
GY	Mg ha^-1^	4505	377.3	2836–5624	12.9	7.28	31.4	0.69
PH	cm	100.4	11.28	61.00–121.9	67.0	NA^§^	33.0	0.80
MD	days	134	4.07	121–141	76.3	5.49	8.94	0.94

^†^Standard deviation.

^‡^G, soybean genotype; GxE, genotype-by-environment interaction.

*H* ^2^ -- Broad-sense heritability on an entry-mean basis.

^§^Plant height was measured at only one location.

**Figure 3:**
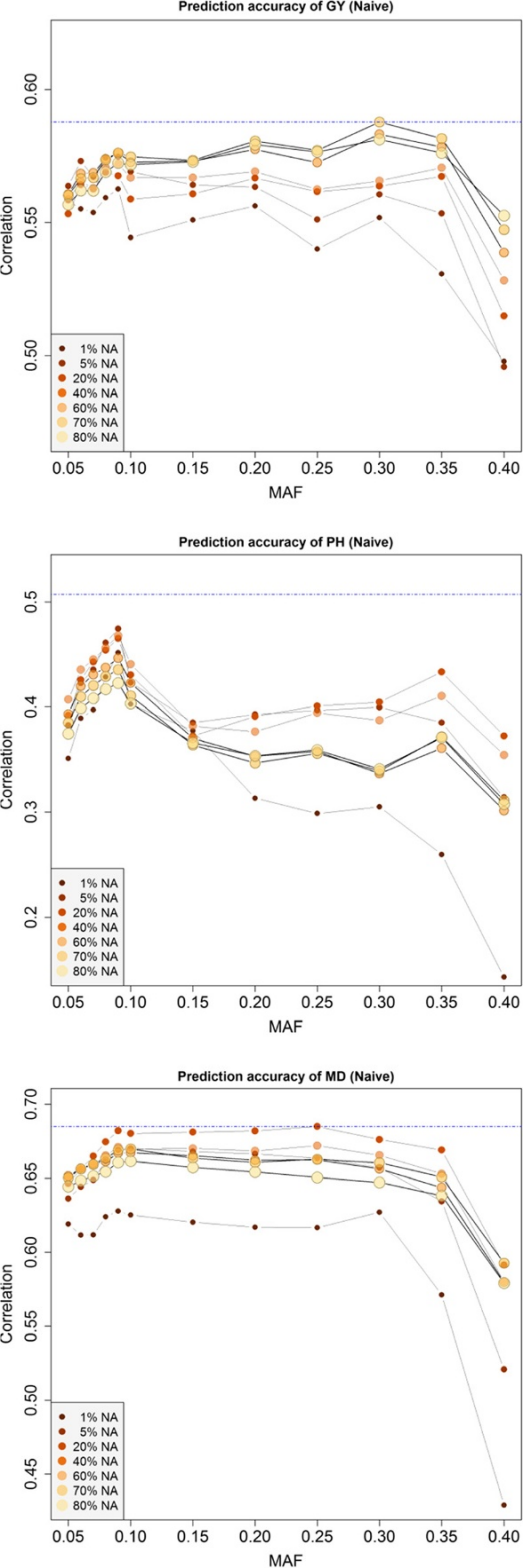
**Average predictive abilities (y-axis) at each combination of minor-allele frequency (MAF) and percent missing value (PMV) for grain yield (GY), plant height (PH) and days to maturity (MD).** Naïve imputation was used to fill in missing values.

The effect of SNP filtering on  was assessed by building a series of G-BLUP models using SNP datasets created by applying combinations of MAF and PMV filtering criteria. Number of SNPs is quickly reduced as SNPs are filtered based on MAF and PMV (Figure 2). Overall, marker filtering criteria did not have a large effect on  for GY, but some important effects were observed for PH and MD (Figure 3). For PH,  was greater when markers with MAF between 0.08 and 0.10 were used compared to all other MAF cutoffs. When considering jointly both filtering criteria, the  of a trait were maximized at different combinations between PMV and MAF. For GY the maximum  (0.59) was obtained with a marker dataset including SNPs with up to 80 PMV and MAF greater than 0.30. For PH and MD,  was maximized when only SNPs with lower PMV were included (Figure 3).

### Imputation

**Figure 4:**
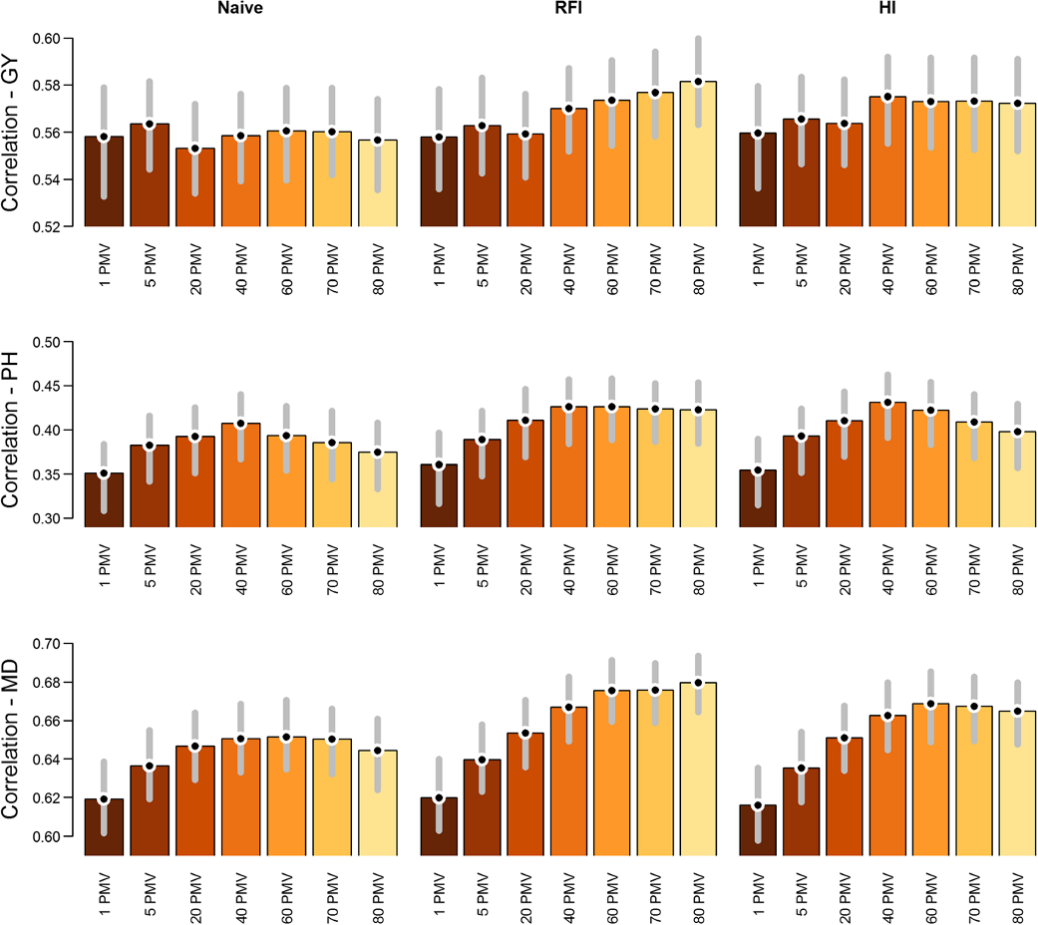
**Average predictive ability and corresponding 95% bootstrap confidence intervals for multiple levels of percent missing values (PMV) and three imputation methods: Naïve, random forest imputation (RFI), and haplotype-based imputation (HI).**

No significant differences in  were found among the imputation methods (Figure 4). When Naïve imputation was used,  was slightly reduced by including SNPs with high levels of PMV. When random forest imputation was used, however,  was maximized when all SNPs were included in the model. A random forest imputation with 80 PMV provided the highest  overall (Figure 4). The random forest method provided numerically higher  than the HI method at high PMV levels, but differences were not statistically significant.

### Model comparison

Contribution of polygenic additive-by-additive epistatic interactions to total phenotypic variation was assessed by constructing epistatic relationship matrices using the Hadamard product of the additive relationship matrix [23], as well as the marker-generated additive-by-additive relationship matrix as described by Xu [24]. For these comparisons, a marker set including SNPs with PMV ≤ 80% and MAF > 0.05 was used. Missing values were imputed using the Naïve method.

The percentages of phenotypic variation accounted for by each model term varied across traits. For GY, the realized additive relationship matrix captured 91.2% of total phenotypic variance. Since the **G**°**G** and **K**_aa_ matrices are highly collinear with **G** (data not shown), the epistatic relationship matrices accounted for similar amounts of variation (Table 3). When combining both additive and epistatic effects into the same model, the epistatic effects account for variable amounts of phenotypic variation. Nevertheless, the percentage of residual variation is fairly constant (Table 3), indicating that including an additive-by-additive epistasis relationship matrix provides no improvement over standard additive G-BLUP models. This was also observed using the cross-validation approach to evaluate . No difference was observed among the models regarding  (Table 4).

**Table 3:** **Percentage of phenotypic variation in grain yield (GY), plant height (PH), and days to maturity (MD) explained by additive and non-additive effects included in models 1 – 5**

Model	Percentage of phenotypic variance accounted for by each component											
	GY				PH				MD			
	G	G°G	K _aa_	Res	G	G°G	K _aa_	Res	G	G°G	K _aa_	Res
[1] G	91.2			8.8	91.8			8.2	94.2			5.8
[2] G°G		86.7		13.3		86.4		13.6		90.0		10.0
[3] K_aa_			86.9	13.1			86.3	13.7			90.1	9.9
[4] G_G°G	49.0	39.7		11.3	73.7	16.3		9.9	28.7	62.2		9.1
[5] G_K_aa_	69.7		19.9	10.4	74.7		15.9	9.5	49.1		42.9	8.0

**Table 4:** **Predictive abilities for grain yield (GY), plant height (PH) and days to maturity (MD) under models [1] – [5]**

Model	GY	PH	MD
[1] G	0.60	0.45	0.67
[2] G°G	0.58	0.43	0.68
[3] K_aa_	0.58	0.43	0.68
[4] G_G°G	0.59	0.45	0.68
[5] G_K_aa_	0.59	0.44	0.68

### Training population size

**Figure 5:**
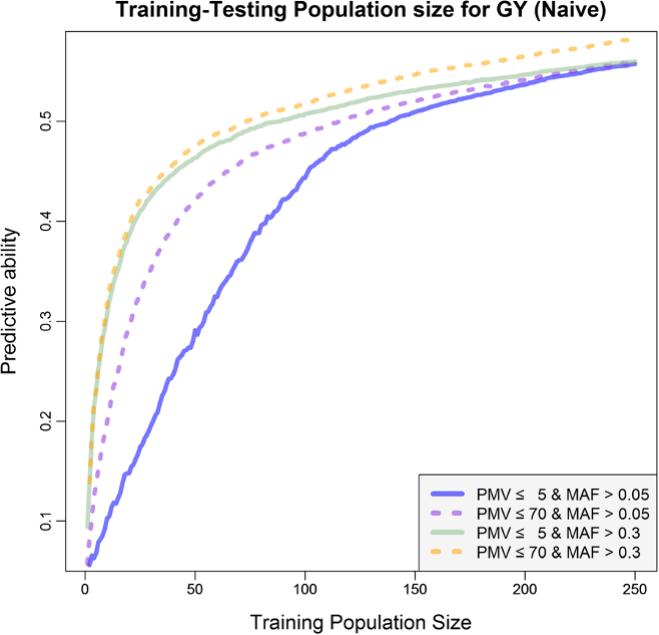
**Relationship between predictive ability and training population size for multiple levels of percent missing values (PMV) and minor-allele frequency (MAF).** The trait displayed here is grain yield (GY).

For a set of SNPs with PMV ≤5% and MAF > 0.05,  plateaued around a training population size just greater than 100 (Figure 5). Predictive ability, however, did steadily increase up until the maximum training population size possible in the cross validation strategy. Predictive ability was improved at lower TP sizes only when a MAF > 0.30 was used to construct **G**. For example, using a MAF > 0.05 and TP size of 50,  was only 0.28, but when a MAF > 0.30 was used,  was increased to 0.47. A TP consisting of at least 100 individuals was required to reach this  using SNPs with MAF > 0.05 (Figure 5).

## Discussion

The results of this study suggest that the use of GBS for genomic selection holds good potential for improving soybean grain yield. Using a cross-validation approach, genomic predictions explained ~32% of the variation in yield phenotypes. After using the phenotype heritability to correct for random environmental deviations included in the validation phenotypes, approximately 41% of the variation in genotypic values was explained by genomic predictions. Because validation phenotypes (i.e., soybean line means) include both additive and non-additive effects, and genomic predictions using the G-BLUP model include only additive effects, this estimated prediction accuracy is likely biased downward.

In order to quantify the relative benefit of genomic selection over phenotypic selection, Technow et al. [32] rearranged the formula for relative response to indirect selection to obtain the inequality , where *L*_Y_ is the cycle length of genomic selection, *r*_A_ is the genomic prediction accuracy, *H*_X_ is the phenotypic selection accuracy, and *L*_X_ is the cycle length of phenotypic selection [32]. Substituting the values estimated herein for grain yield into this formula indicates that genomic selection is expected to be superior to phenotypic selection in terms of genetic gain per unit time if the cycle length of genomic selection is less than 77% the cycle length of phenotypic selection. It is entirely possible for a genomic selection cycle to be 66% of a phenotypic selection cycle based on the structure of the University of Nebraska Soybean Breeding Program. On top of this, the above formula assumes equal selection intensities for genomic and phenotypic selection. As genotyping costs continue to decline, selection intensity for genomic prediction could be increased compared to phenotypic selection at equal cost. Before soybean breeding programs incorporate genomic selection on a large scale, these results need to be validated through comparisons of phenotypes and genomic predictions across years, as well as by comparison of progenies from phenotypic and genomic selection programs.

The high genomic prediction accuracy observed was fairly consistent across SNP datasets with differing levels of PMV. More than 16,000 SNPs were scored with less than 5 PMV using GBS, which is surely more than is needed to ensure good SNP-QTL linkage disequilibrium among elite soybean breeding progenies [33]. Little to no sacrifice in accuracy was observed when SNPs with up to 80 PMV were included. It might be desirable to reduce the SNP numbers to ease computational requirements when predicting individual SNP effects and summing effects to calculate genomic predictions. However, more saturated SNP datasets may be more desirable for computing genomic predictions of multi-family selection schemes of more diverse germplasm. The G-BLUP approach is more computationally efficient with computational demands scaling with individual number rather than marker number. Knowing that data filtering steps are not likely needed for using GBS data for genomic prediction reduces the number of optimization steps and simplifies the process.

We failed to find significant differences among imputation methods, including differences between Naïve imputation and the other two which use covariance information between nearby SNPs. While not significantly better, the machine learning, non-parametric method called random forest performed best when SNPs with up to 80 PMV were included. This was also observed by Rutkoski et al. [18], but these authors did not compare random forest imputation with a method using marker order information. We observed that a haplotype-based method, which used marker order information from the soybean physical map, was not superior to random forest imputation. Random forest has often been used for imputing markers for genomic selection in plant breeding, especially when a reference genome is not available [10, 15, 18]. A haplotype-based method was also used for GBS data by Crossa et al. [16], but these authors also observed very little to no advantage over Naïve imputation. This general lack of benefit to imputation is likely due to the fact the genomic prediction is robust to missing marker data [18] and the number of markers with relatively low PMV provided by GBS is more than enough to cover the linkage disequilibrium space in crop breeding germplasm.

Rather than compare shrinkage models and Bayesian variable selection models for prediction accuracy as has been frequently performed previously (e.g., [10, 21]), we compared G-BLUP models including additive effects only against those also including additive-by-additive effects. Additive-by-additive interaction effects were incorporated into the model in the Cockerham-Kempthorn fashion by including a random polygenic interaction effect with a covariance structure specified as the Hadamard product of the additive genomic relationship matrix [34]. This model makes many assumptions, and the soybean population clearly violates the assumptions of linkage equilibrium between loci and randomly mating individuals. Because of this violation, another formulation of the additive-by-additive relationship matrix according to Xu [24] was used. It turned out the **K**_aa_ matrix calculated according to Xu [24] was highly collinear with the simple Cockerham-Kempthorn Hadamard product and explained similar amounts of phenotypic variation. Neither **G** ∘ **G** nor **K**_aa_ was orthogonal to the **G** matrix as can be seen by the variance component estimation. Similar amounts of variation were explained when any of these effects were included in the model alone or together. The amount of residual variation was actually slightly smaller when only **G** was modeled and genomic prediction accuracies were not enhanced by including additive-by-additive effects using either the Cockerham-Kempthorn or Xu [24] formulation.

Low to moderately sized training populations could be used in a soybean breeding program to achieve adequate prediction accuracies (Figure 5). Although it’s probably not necessary to reduce TP sizes down to such a low level, training population sizes could be reduced further if only SNPs with higher MAF are included. The underlying reason for this is not clear. It is possible that SNPs with low MAF are not sampled by chance when small training populations are sampled and phenotyped. If they are not polymorphic in the TP, they cannot contribute information to the relationships between individuals, which is the basis of predictions in G-BLUP. When TP size is increased, SNP alleles with low frequency are more likely to be adequately represented in the TP. When MAF threshold is higher, this problem is reduced by the fact that all SNP alleles have a reasonable chance of contributing to relationship even when TPS sizes are small.

## Conclusions

This first look at GBS for genomic prediction in soybean suggests GBS holds good potential to enhance genetic gain in soybean. Over 16,000 SNPs were scored with less than 5% missing data. Filtering markers based on amount of missing data had little to no effect. No differences were observed among imputation methods. The highest accuracies were observed when random forest imputation was used on all SNPs, but differences were not significant. A standard additive G-BLUP model was robust; modeling additive-by-additive epistasis did not provide any improvement in prediction accuracy.

## Abbreviations

MAF: Minor-allele frequency
PMV: Percent missing values
RFI: Random forest imputation
MAS: Marker-assisted selection
GS: Genomic selection
G-BLUP: Genomic best linear unbiased prediction
*r*_PP_: Predictive ability
GY: Grain yield
PH: Plant height
MD: Days to maturity
SCN: Soybean cyst nematode
GBS: Genotyping by sequencing
SNP: Single nucleotide polymorphism
HI: Haplotype-based imputation.
